# Dr. Ferguson on the Nature and History of Marsh Poison

**Published:** 1821-12-01

**Authors:** 


### VIII.

*On the Nature and History of Marsh Poison.* By William Ferguson, M. D. F. R. S. E. Inspector of Army Hospitals. (From the Transactions of the Royal Society of Edinburgh.) Quarto, pp. 29. Edinb. 1821.

---

——Quod sol atque imbres dederant quod terra crearat,
Sponte sua. Lucret.

---

It is nearly correct that one half of life is spent in *unlearning* what was taught us during the other half. This melancholy truth is peculiarly applicable to many parts of medical science, where opinions, apparently based on facts, are revolutionized with astonishing, and sometimes whimsical rapidity. We need go no farther than the subject of fever, as far as regards its nature, cause, and treatment, to illustrate the above assertion. The warfare between the contagionists and anti-contagionists is active on both sides of the Atlantic; but the former class have most power over the minds of the community at large, whose fears and prejudices preponderate, with overwhelming force on the side of contagion.

One point of the etiology of fever, however, seems to have long rested on a solid base—namely, the agency of vegeto-animal, or, as it is usually termed, marsh miasma, on the human constitution. All conjectures, indeed, respecting the essence or nature of this invisible agent, have subsided, for we now begin to feel a conviction, that the essences of things are beyond our ken. But a great many of the laws which govern, and the consequences produced by marsh effluvium, were supposed to be understood. A very general (but not universal, as Dr. Furguson supposes) opinion prevailed, and still prevails, that the agent in question owes its deleterious influence to "vegetable or aqueous putrefaction,"—an opinion which it is Dr. Ferguson's object to prove *unfounded*—"because, as. will presently be seen, the marsh must cease to be a marsh, in the common acceptation of the word, and the sensible putrefaction of water and vegetables must alike be impossible, before its surface can become deleterious." It will also be seen, he observes, that a healthy condition of soil, in pestiferous regions, "is infallibly regained by the restoration of the marshy surface in its utmost vigour of vegetable growth and decay." It is very difficult to reconcile this assertion with what hundreds now living saw at Walcheren in 1809, (not 1810 as Dr. F. states) where the greatest possible degree of sickness prevailed in those parts, especially round Flushing, where the ground was *half inundated*, and consequently where growth and decay were going forward in vigour. We do not mean to argue, indeed, that dry soils and seasons must consequently be healthy ones. We know how often it is otherwise; but still we believe that, speaking generally, that soil is in the best condition for giving out miasmata, which is just in a state neither dry nor overflowed, but exposing a slimy vegetable, muddy or miry surface, to the action of an autumnal sun. Speaking of Rosendaal, Dr. Furguson observes, that it was covered with stunted heath plants, and that, "on digging, it was universally found to be percolated with water to within a few inches of the surface, which, so far from being at all putrid, was perfectly potable in all the wells of the camp." Now we do not think there is any incongruity in supposing the water to be good when below the surface, but capable, in its ascent through the heath plants, of carrying or causing those effluvia which experience shows to be so deleterious to life. We know nothing of the taste, smell, or sensible properties (if at all sensible) of marsh miasma, and therefore we are not authorised to say that water is divested of them because it is clear or potable. Indeed we have strong reason to believe that water, in the form of rain, dews, or even collected in wells and cisterns, in unhealthy climes, is often impregnated with what we term marsh effluvium. Still, however, a cloud of mystery hangs over the production and extrication of this febrific agent; for in the same place, and apparently in similar years as to temperature, rains, &c. we shall find the inhabitants and sojourners at one time healthy, and at another sickly.

Dr. Nicholl, a physician of great intelligence and discernment, inspector of hospitals on the coast of Africa, has pourtrayed the capricious and uncertain generation of fevers there, in very striking colours, but we have not room to introduce his observations in this place.

Dr. Ferguson proceeds to state several interesting particulars relative to the medical topography of those countries through which he has campaigned.

In the month of June 1809, our army marched through a singularly dry, rocky, and elevated country, on the confines of Portugal, the weather having been previously so hot, for several weeks, as to dry up the mountain streams. "In some of the hilly ravines, that had lately been water-courses, several of the regiments took up their bivouac, for the sake of being near the stagnant pools of water that were still left among the rocks." Several men were seized with remittent fevers before they could leave the bivouac next morning, and that type of fever continued to affect the portion of troops exclusively which had so bivouacked, for a considerable time. This incident is adduced by our author to prove that the "humid decay of vegetables" is not essential to the production of pestiferous miasmata. But we confess that we see no proof of the non-existence of decaying vegetable and animal substances, when we are bivouacked in the bed of a "half-dried ravine," and near "stagnant pools of water." If we examine narrowly into the state of things, we shall scarcely find a spot of this earth's surface that is not covered or embued with both vegetable and animal remains, in a state of decomposition; and ready to afford pabulum for the sun's rays, with or without humidity, to extricate the injurious principle in question. Nor do we see any thing in the following passage to contravene, but much to confirm, what we have here advanced.

"The army advanced to Talavera, through a very dry country, and in the hottest weather fought that celebrated battle, which was followed by a retreat into the plains of Estremadura, along the course of the Guadiana river, at a time when the country was so arid and dry, for want of rain, that the Guadiana itself, and all the smaller streams, had in fact *ceased to be streams*, and were no more than *lines* of *detached pools* in the courses that had formerly been rivers; and there they suffered from remittent fevers of such destructive malignity, that the enemy, and all Europe believed, that the British host was extirpated; and the superstitious natives, though sickly themselves, unable to account for disease of such uncommon type amongst the strangers, declared they had all been poisoned by eating the mushrooms, (a species of food they hold in abhorrence,) which sprung up after the first autumnal rains, about the time the epidemic had attained its height. The aggravated cases of the disease differed little or nothing from the worst yellow fevers of the West Indies; and in all the subsequent campaigns of the Peninsula, the same results uniformly followed, whenever, during the hot season, any portion of the army was obliged to occupy the arid encampments of the level country, which at all other times were healthy, or at least unproductive of endemic fever." P. 5.

Those who have travelled through, or resided any time in Sicily, are familiar with circumstances of the above description, of which Irvine and Boyle have related many particulars. From the former of these authors we shall make an extract illustrative of the sentiments here advanced.

"Sicily is penetrated in several directions by ridges of primitive hills of considerable height: between these are numerous water courses, which are dry in summer, and occasionally filled by torrents in winter. They are designated by the Sicilians, FIUMARI, and are used as roads in the dry season. Many of them are extremely unhealthy in the latter part of summer, and in autumn, and infested by what the natives term MALARIA. The state of this *Malaria* varies much according to the state of the season. A very wet season will *overwhelm*, as it were, the sources of this febrific, while a very dry one will so parch up the surface of the earth, as to produce a similar effect. At LENTINI, however, around which the country is marshy, with a considerable lake in the vicinity, the ground is *partly* freed from water in hot weather, but is never so dry as to prevent the formation of miasmata. Here there is a Malaria every year. In many of the *fiumares* the stream disappears in the gravel, and percolates under the surface to the ocean. Thus at the bottom of the large *fiumare* which bounds Messina on the northern side, fresh water will be found at a foot depth close to the sea. It is in these kinds of fiumares that a Malaria prevails, according to the opinion of the natives, throughout the year; and this probably accounts for the extrication of miasmata in many parts of the West Indies as well as Europe, where there are apparently no materials for their production. Thus some places in Sicily, though on very high ground, are sickly; as Ibesso or Gesso, about eight miles from Messina, situated upon some *secondary* mountains lying on the side of the primitive ridge which runs northward towards the Faro, which has always been found an unhealthy quarter for English troops. It stands very high; but still there is higher ground at some miles distance. Water is scarce here, and there is nothing like a marsh.—At this station, however, sickness seldom occurs "unless after rains falling while the ground is yet hot, that is, during the heat of summer, or early in autumn, when all circumstances combine for the production of miasmata." *Irvine*, p. 6.

The medical topography of Lisbon and vicinity is interesting. The breadth of the Tagus, at this capital, is not more than two miles—but it is the boundary betwixt sickness and salubrity. The villages and hamlets scattered on the south or Alentejo side of the river—a soil dry superficially, being perfectly flat and sandy—are the most pestiferous abodes. The sickly tract is not confined to the immediate borders of the Tagus. Salvatera, a large village about a mile inland, though reputed healthy till the beginning of autumn, is then deserted by every one who has the means of escape.

"In their superstitious fear, the inhabitants declare, that even the horses and other animals would be seized with fever if left behind, and therefore they always remove the royal stud. The country around is perfectly open, though very low, and flooded with water during the whole of the rainy season; but at the time of the periodical sickness it is always most distressingly dry; and exactly in proportion to the previous drought, and consequent dryness of soil, is the *quantum* of sickness. I have visited it upon these occasions, and found it the most parched spot I ever saw. The houses of the miserable people that were left behind being literally buried in loose dry sand, that obstructed the doors and windows." 6.

Dr. Ferguson adduces another example of this kind near Ciudad Rodrigo, situated on a rocky bank of the river Agueda, a remarkably clear stream. The approach to this town is through "a bare open hollow country, that has been likened to the dried-up bed of an extensive lake. Upon more than one occasion, when this low land, *after having been flooded in the rainy season*, had become as dry as a brick-ground, with the vegetation utterly burnt up, there arose fevers to our troops, which, for malignity of type, could only be matched by those before mentioned on the Guadiana."

We do not think that the above fact disproves the existence of decayed vegetable remains acted on by humidity. We see that the malaria is always *after* the rainy season, and when the surface of the soil is acted on by a powerful sun. We all know too, that in the hottest season there is the most copious precipitation of dew at night, followed by its exhalation in the day. Who is prepared to say that these exhalations of humidity carry with them no noxious miasmata from decayed vegetable and animal remains? There is, we think, much more in favour of this opinion than against it.

During the years 1815-16 and 17, our author was employed in making a medico-topographical survey of the West India Islands—a service that afforded him diversified opportunities of improving the observations he had elsewhere made on the subject under discussion. Dr. Ferguson very truly remarks that—

> "It might *there* be seen, that the same rains which made a deep marshy country perfectly healthy, by deluging a dry well cleared one, where there was any considerable depth of soil, speedily converted it, under the drying process of a vertical sun, into a hot-bed of pestiferous miasmata." 8.

It has always been remarked that a morass or fen, when completely overflowed, becomes harmless, as exemplied in the case of the unwholesome town of Castries, at the bottom of the carenage, in the Island of St. Lucia—a town embosomed in a deep mangrove fen. It became perfectly healthy under the periodical rains; while the garrison on the hill of Morne Fortunée, immediately above it, began to be affected with remittent fevers. The following passage is surely against Dr. Ferguson's doctrine.

> "The top and shoulders of the hill had been cleared of wood, and during a continuance of dry weather, the garrison had no source of disease within itself, but this was amply, though but temporarily supplied, as soon as the rains had saturated the soil on which it stood." 9.

Is not this a proof that it is the exhalation of moisture, saturated with some noxious principle—and what can afford this principle but vegeto-animal decomposition—which is the cause of disease? Dr. Ferguson observes immediately afterwards—that "an uncommonly rainy season at Barbadoes, seldom failed in that perfectly dry and well-cleared country, to induce, for a time, general sickness," while Trinidad, the centre of which may be called "a sea of swamp," was always rendered more sickly by a cessation of the preserving rains. What can be more corroborative of the principle we maintain than the foregoing facts—and still more so the following:—

> "General dryness of soil, however, is far from being the ordinary characteristic of our West India colonies. The swamp is too often exposed to the continued operation of a tropical sun, and its approach to dryness is the harbinger of disease and death to the inhabitants in its vicinity." 9.

Dr. Ferguson observes, what we have occasionally seen, that an offensive odour is by no means a certain indication of uniform insalubrity in a marsh. Thus the town of Point au Petre, in Guadaloupe, is situated among the most putrid marshes in the world, the stench of which is never absent from the streets—yet the place was far from being *uniformly* unhealthy. Strangers, though much annoyed by the smell, often resorted to the place with impunity.

" But at Fort Fleur d'Epeé, the farthest out-post, at the extremity of the marshes, where they approach to the state of Terra Firma, where little or no water is to be seen on the surface, and no smell exists, there cannot be supposed a more deadly quarter, and all white troops considered their being sent there, as equivalent to a sentence of death." 10.

It has long been known that the *lower* apartments of a building, situated in a place capable of giving out these deleterious miasmata, were always more unhealthy than the *higher*. This will not hold good with respect to elevated grounds in the *vicinity* of a marsh. These elevations would appear to attract the deleterious principles floating in the atmosphere in the manner they attract the clouds and rains. Thus Port d'Espagne, Trinidad, is situated close to the great eastern marsh; and although not a healthy town, it is not uninhabitable. On the right are some heights which rise out of one extremity of the marsh. These are composed of pure limestone, and have proved a residence deadly and destructive, in the highest degree. " No place, however elevated, or sunk, or walled in, or sheltered, gives security against the exhalations from below." It has been distinctly ascertained, indeed, that the degree of insalubrity is in proportion to the degree of elevation. The summit, 400 feet above the level of the marsh, is so deadly a spot, that not even a Creole Mulatto Spaniard could sleep in it with impunity, for a single night, after a course of dry weather.

Another curious example is given at the beautiful post of Prince Rupert's, Dominica, which is a peninsula comprehending two hills of romantic form, joined to the main land by a flat marshy isthmus to windward. The two hills jut out on the same line into the sea, the inner hill being a pyramid 400 feet high above and across the marsh—the outer hill forming a bluff promontory overhanging the sea. Between these is a narrow clean valley, which was pitched on for a garrison establishment, but found unhealthy. A barrack was then constructed on a natural recess or platform on the inner hill, 300 feet above the marsh; "but it proved to be pestiferous beyond belief—in fact, no white man could live there." A quarter was built on the *outer* hill, on nearly the same line of elevation, and exactly 500 yards farther from the swamp. This was found perfectly healthy.

This curious fact proves, we think, incontestibly, that the swamp was the source of deleterious effluvium, and that the localities, terrestrial and atmospheric, determined the current of this noxious exhalation on the position taken up on the inner hill.

The following piece of medical topography will be read with much interest.

"In the Island of Antigua, the same results were confirmed in a very striking manner. The autumn of 1816 became very sickly, and yellow fever broke out in all its low marshy quarters, while the milder remittent pervaded the island generally. The British garrison of English Harbour soon felt the influence of that most unwholesome place. They were distributed on a range of fortified hills that surround the dock-yard. The principal of these, Monks Hill, at the bottom of the bay, rises perpendicular above the marshes to the height of 600 feet. The other garrisoned hill, which goes by the name of the Ridge, is about 100 feet lower, but instead of rising perpendicularly, it slopes backwards from the swamps of English Harbour. It was the duty of the white troops, in both these forts, to take the guards and duties of the dock-yard amongst the marshes below, and so pestiferous was their atmosphere, that it often occurred to a well-seasoned soldier mounting the night-guard in perfect health, to be seized with furious delirium while standing sentry, and when carried to his barracks on Monks Hill, to expire in all the horrors of the black vomit, within less than 30 hours from the first attack; but during all this, not a single case of yellow fever, nor fever of any kind, occurred to the inhabitants of Monks Hill; that is to say, the garrison staff, the superior officers, the women, the drummers, &c. all in fact that were not obliged to *sleep* out of the garrison, or take the duties below, remained in perfect health. The result on the Ridge was not quite the same, but it was equally curious and instructive. The artillery soldiers, (17 in number) never took any of the night guards, but they occupied a barrack about 300 feet above the marshes, not perpendicular above them, like Monks Hill, but a little retired. Not a case of yellow fever or black vomit occurred amongst them, but every man, without a single exemption, suffered an attack of the ordinary remittent, of which one of them died; and at the barrack on the top of the Ridge, at the height of 500 feet, and still further retired from the marshes, there scarcely occurred any fever worthy of notice." 14.

Another property of the marsh poison, is its attraction for, or adherence to, lofty umbrageous trees. In the territory of Guiana particularly, where these trees abound, "it is wonderful, says Dr. F. to see how near *to leeward* of the most pestiferous marshes, the settlers will venture with impunity to place their habitations, provided they have this security."

"The town of New Amsterdam in Berbice, is situated within short musket-shot to leeward of a most offensive swamp, in the direct tract of a strong trade-wind, that blows night and day, and pollutes even the sleeping apartments of the inhabitants with the stench of the marshes; yet it brings no fevers, though every one is well aware, that it would be almost certain death for an European to sleep, or even to remain after night-fall, under the shade of the lofty trees that cover the marsh, at so short a distance. All, too, are equally aware, that to cut down the trees would be a most dangerous operation in itself, and would certainly be productive of pestilence to the town." 14.

From these and various other facts our author draws two principal conclusions. 1st. That the marsh poison has no connexion with *the putrefaction* of vegetable substances—a conclusion to which we cannot entirely subscribe, for the reasons before stated;—and 2dly, that the marsh poison cannot arise from simple humidity, in which we quite agree with our able and experienced author. We also agree with Dr. Ferguson, that the febrific effluvium has its principal source in the half-dried or drying margins or other points of swamps, and that complete inundation is generally a safeguard against the exhalation.

"One only condition then seems to be indispensable to the production of the marsh poison, on all surfaces capable of absorption; and that is, the *paucity* of water, where it has previously and recently *abounded*. To this there is no exception in climates of high temperature; and from thence we may justly infer, that the poison is produced at a highly advanced stage of the *drying* process;—but, in the present state of our knowledge, we can no more tell what that precise stage may be, or what that poison actually is, the developement of which must necessarily be ever varying, according to circumstances of temperature, moisture, elevation, perflation, aspect, texture, and depth of soil, than we can define and describe those vapours that generate typhus fevers, small pox, and other diseases. The marsh and the stagnant pool will no doubt be pointed out as the ostensible sources from which this poison has ever sprung; but the marsh, it has been seen, is never pestiferous when fully covered with water. At all other times it must present a great variety of drying surface, and both the lake and the marsh must ever possess their saturated, half-dried, and drying margins." 18.

Dr. Ferguson concludes this part of the subject with some remarks on other properties of marsh poison, besides those already noticed. He thinks there are no experiments yet made which determine whether the poison be specifically lighter or heavier than common air; but it evidently possesses much attraction for the earth's surface. The official accounts of the last sickly season at Barbadoes shewed two thirds more sickness on the ground floors than in the upper stories of the barracks.

Dr. F. thinks it a proof of the attraction abovementioned, that the malaria creeps along the ground, so as to concentrate and collect on the sides of the adjacent hills, rather than float directly upwards in the atmosphere. Another remarkable fact is, that it seems to be lost or absorbed by passing over a small surface of water. "The rarifying heat of the sun, too, certainly dispels it, and it is only during the cooler temperature of the night that it acquires body, concentration, and power." Currents of air also dissipate this poison; and our author thinks that the West India Islands would be uninhabitable, were it not for the trade winds. Where this salutary breeze is interrupted through circum stances of season, or intervention of high hills, the consequences are most fatal. The leeward shore of Guadaloupe, for a course of nearly 30 miles, under the shelter of a very high steep ridge of volcanic mountains never felt the sea breeze, nor any but the night land-wind from the mountains; and though generally dry, and devoid of marshes, it is inconceivably pestiferous throughout the whole tract.* The same remark applies to the greater part of the leeward coast of Martinique—indeed to the leeward alluvial basis of hills, in whatever part of the torrid zone they may be situated, with the exception perhaps of the immediate sites of towns, where the pavements prevent the rain-water being absorbed into the soil.

As to remedy for malaria, if there *be* one, our author thinks "it must be found in the powers of cultivation, ever opening the surface for the escape of pestilential gases, and exhausting the morbific principles by a constant succession of crops."

---

* "The point of Dungeness, on the coast of Kent, is a tongue of land appended to the great Romney marsh, and consists of an extensive bank of shingle or gravel, so dry, loose, and open, that even in dry weather, horses sink in it nearly up to the knees. The forts and barracks are about four miles from the main-land, where grass begins to grow; yet there was no spot of that unwholesome tract of country more prolific of endemic fever, during the hot summer and autumn of 1807, than this station." P. 21.

"For wherever malaria prevails, the uncultivated savannah, even though used for pasture, becomes infinitely more pestiferous than the plantation, and the depopulated country falls completely under its dominion. With the aid of the purifying sea-breeze, this course at the British colony of Demerara, within six degrees of the equator, has succeeded in rendering the cultivated portion of the deepest and most extensive morass probably in the world, a healthy, fertile, and beautiful settlement." 22.

Dr. Ferguson thinks, and we agree with him, that the marsh poison has been falsely accused of producing other distempers, as dysentery for instance, because they happen to coexist with it. He thinks it produces but its own specific disease, varying, however, in form, from the common ague of Lincolnshire, through all the milder remittent types, up to the aggravated yellow fever, or malignant remittent of the West Indies—"and *that* variation so certain and uniform, in proportion to the remote exciting cause, that the varying types of fever might be measured almost to a certainty by the degrees of solar heat, as marked on the thermometer." Thus it is as rare to meet with ague on the swampy alluvial plains of the West Indies, as it is to find any thing else but a common remittent or intermittent on the cooler mountain marshy levels of the same country.

The professional world is aware how decidedly anti-contagious is Dr. Ferguson's creed. Although we are not inclined to go the lengths which our author goes, on this much litigated question, yet we deem it right to extract a note which he has appended to his interesting paper on marsh poison, as declaratory of his statement.

"The yellow fever cannot be a contagious disease, *because*, during its utmost rage, it is confined almost exclusively to a particular and very limited class of the inhabitants of the West Indies, viz. the newly arrived; and never affects the coloured people, unless it finds them under the same circumstances, of being newly arrived from a cold climate; although *that last class* is the most numerous, by at least ten to one, of the inhabitants, and is besides the most liable, of all mankind, to fall under the influence of every acknowledged contagion, such as Typhus Fever, Plague, Small Pox, Measles, and Scarlatina.

"It cannot be a contagious disease, *because*, even amongst white people, it has been proven from official returns, that the attendants on the sick are less liable to be attacked with fever than those who have never approached the sick-bed, and because it has also been proven, in a multiplicity of instances, that the disease is not communicable to the wounded, the surgical sick, the convalescent, and the healthy, though occupying the most contiguous beds in the same hospital.

"It cannot be contagious, *because* it has also been frequently seen, that when a regiment has been divided into separate detachments, the different divisions have been affected with distinct types of fever, according to the circumstances of temperature and locality of their respective quarters; and when one of them happened to be stationed in the locality of yellow fever, (*which is always at or near the level of the sea,*) that form of fever was incapable of being conveyed to the other detachments in the higher ranges of country, however frequent and indispensable may have been the necessary communication between them.

"It cannot be contagious, nor any thing but a seasoning remittent fever, of violent and malignant form, peculiar in a great degree to the newly arrived, *because* all who have been debilitated by long residence in hot climates, and would therefore be the first to fall under the influence of a new plague, are in a great degree exempt from this form of the disease. And, lastly, it cannot be contagious, nor any thing but the product of unwholesome locality, and uncommon drought of season, *because*, in the warmer countries of Europe and North America, where all the inhabitants are under the same circumstances as the newly arrived in the West Indies, from the effect of the preceding winter, it has never been seen except in some particular low situations, where the heat has been steadily, for a considerable time previously, of the West India temperature; nor retained in them after that degree of heat has been changed by the change of season, nor transported from them, even during its utmost rage, to other localities in the closest vicinity, if of higher elevation, of better ventilation, and cooler atmosphere.

"The foregoing are not *vague assertions*, but matters of *fact*, that have been verified and recorded by the official returns of our armies in the West Indies for the last twenty-five years.

"As in every epidemic, where multitudes are in the course of being affected, every supposable degree of communication must of necessity be constantly taking place amongst the inhabitants of a crowded camp, or city; all or any of the believers in contagion, may have their creed confirmed in any manner they please, from the dead or the living, by the passing events of every day; and it is only by reference to such facts as the above that the delusion can be cured, and that the observer can be brought to distinguish clearly between the agency of *epidemic* and *contagious* influence. Those, however, who have only *read* the reports of panic, from the theatre of the epidemic, will seldom be cured of the delusion; no more will those who have *seen* the disease, but have fled in affright from its supposed contagion; but all who are compelled to *remain* within its epidemic current, and *witness* the progress of its successive invasions, through the recurrence of sickly seasons, must infallibly have their eyes opened to its real nature, if they be at all capable of distinguishing truth from error.

"In opposition to the fact that has been so often verified in every colony of the West Indies, that the sailors of merchant ships landed with yellow fever, never infected the crowded, unwholesome suburblodging houses, to which alone they had access, it has been said, with much feasibility, to have been *imported* in ships. But this is another delusion, arising from the well-known fact, that newly arrived strangers are generally the immediate and most striking victims of every epidemic; and hence our most thoughtless intemperate sailors, when at these dangerous times they are thrown into the unwholesome anchorages of the West Indies, are not only the first to suffer from the epidemic in its course, or about to begin, but they are denounced as the *importers* by the prejudiced vulgar; and the accusation is loudly echoed, even among the better informed, by all who wish to make themselves believe, that pestilence cannot be a native product of their own habitations. The incomprehensible punctuality of ships regularly arriving at some particular sea-ports of Spain and North America, fraught with the pestilence of yellow fever, at the precise stage and period, *and at no other*, of those hot and dry seasons that assimilate them to the unwholesomest of the West India towns, can therefore be no more than a fiction of prejudice,—a delusion of panic terror." 29.

We consider the profession, and expecially the tropical visitor, as under much obligation to Dr. Ferguson, for the valuable facts and observations contained in the foregoing paper, of which we have offered a more simple analysis than we otherwise should have done, had it been published in a form likely to travel widely through the profession.

